# Effects of lighting conditions and accommodation on the three-dimensional position of Visian implantable collamer lens

**DOI:** 10.1186/s40662-022-00313-2

**Published:** 2022-11-04

**Authors:** Lingling Niu, Zhe Zhang, Huamao Miao, Jing Zhao, Xiaoying Wang, Ji C. He, Xingtao Zhou

**Affiliations:** 1 Department of Ophthalmology and Optometry, Eye & ENT Hospital, Fudan University, Shanghai, China; 2 NHC Key Laboratory of Myopia (Fudan University), Shanghai, China; 3 Key Laboratory of Myopia, Chinese Academy of Medical Sciences, Shanghai, China; 4 Shanghai Research Center of Ophthalmology and Optometry, Shanghai, China; 5 New England College of Optometry, MA Boston, USA

**Keywords:** Implantable collamer lens, Anterior segment optical coherence tomography, Myopia, Tilt

## Abstract

**Background:**

To investigate the effects of lighting conditions and accommodation on the three-dimensional position of Visian implantable collamer lens (ICL V4c).

**Methods:**

This observational study recruited 62 eyes of 31 myopia patients underwent ICL V4c implantation. Anterior segment optical coherence tomography (AS-OCT) assessed the anterior chamber depth (ACD), ACD-ICL (distance from the corneal endothelium to anterior surface of the ICL V4c), vault (distance between the posterior ICL V4c surface and anterior crystalline lens surface), and crystalline lens tilt under various lighting conditions and accommodation relative to the corneal topographic axis at one year after ICL V4c implantation. Baseline was defined as the scotopic condition, which was also the non-accommodative stimulus condition. The ICL V4c tilt was analyzed using MATLAB. The significance level was set at *P* < 0.05.

**Results:**

The ACD-ICL values were similar under various lighting conditions (*P* = 0.978) but decreased during accommodation (*P* < 0.001). The vault was significantly smaller under mesopic and photopic conditions than the baseline (*P* = 0.044 and *P* < 0.001, respectively) but remained unchanged during accommodation (*P* = 0.058). The inferotemporal proportion of ICL V4c (88.7%, 55 eyes) and crystalline lens (74.2%, 46 eyes) tilts were not significantly different (*P* = 0.063). Crystalline lens under various lighting conditions and accommodation exhibited similar tilts. The vertical tilt of ICL V4c was significantly larger under photopic conditions than the baseline (*P* = 0.038). The horizontal and total tilts were significantly decreased during accommodation (*P* = 0.043 and 0.013, respectively).

**Conclusions:**

The axial position of ICL V4c in the anterior chamber was stable under various lighting conditions. Lighting conditions and accommodation may influence vertical, horizontal and total tilts of ICL V4c.

**Supplementary Information:**

The online version contains supplementary material available at 10.1186/s40662-022-00313-2.

## Background

Since its approval by the U.S. Food and Drug Administration in 2005, Visian implantable collamer lens (ICL V4c) implantation has gradually become one of the most important surgical procedures for high myopia correction. ICL V4c has demonstrated high effectiveness, safety, predictability, and stability in long-term results [1–3]. The advantages of ICL V4c include no limitation on corneal thickness, fast visual recovery, stable refraction, reversible surgery, and superior visual quality than that after corneal surgery [4–6].

Studies on axial ICL V4c shifts under various lighting conditions and during accommodation using anterior segment optical coherence tomography (AS-OCT) or ultrasound biomicroscopy (UBM) are of considerable interest [7–10]. However, conventional instruments such as AS-OCT, Pentacam, and UBM cannot measure the three-dimensional position of the ICL V4c in the eye to determine its tilt. Instruments such as OPD-scan [11] have measured the tilt of intraocular lenses (IOL) after cataract surgery, but postsurgical ICL V4c tilt has not been reported.

The new generation AS-OCT (CASIA2, TOMEY, Nagoya, Japan) possesses a 1310 nm laser wavelength, and has the advantages of high axial and transverse resolutions of 10 μm and 30 μm, respectively, high repeatability, and is non-mydriatic. Furthermore, it can automatically measure the tilt of the crystalline lens as a reference to the corneal topographic axis. Calzetti et al.[12] reported that the AS-OCT provided repeatable and affordable measurements of the tilt of IOL. IOL tilt after cataract surgery can cause higher-order aberrations, which may lead to visual function deterioration. Previous research found that the tilt of ICL V4c has a positive effect on subjective visual quality [13].

The ICL V4c haptics, placed in the ciliary sulcus, are in direct contact with the ciliary muscle [8]. As previously reported, the posterior surface of the ICL V4c is not in contact with the crystalline lens, while the anterior surface is attached all around the iris [8]. It is assumed that the external forces on the ICL V4c arise from the ciliary muscle and the iris, which change depending on the lighting conditions and accommodation. Therefore, it is worth exploring the effects of these changes on the three-dimensional position and alignment (tilt) of the ICL V4c.

## Methods

### Patients

The participants were fully informed of the details and potential risks of the procedure and provided written informed consent. This study adhered to the tenets of the Declaration of Helsinki and was approved by the Ethics Committee of the Eye & ENT Hospital, Fudan University (No. 2016038).

In this observational study, we recruited patients who underwent ICL V4c implantation at the Refractive Surgery Centre of Eye & ENT Hospital. The inclusion criteria were as follows: patients aged 20–42 years, those with a stable refractive error (≤ 0.50 D change per year in refractive error for the past two years), minimum anterior chamber depth (ACD) of 2.8 mm, minimum endothelial cell density (ECD) of 2000 cells/mm^2^, and no contact lens use for at least two weeks. The exclusion criteria were as follows: patients with comorbid eye disorders, suspicion of keratoconus and presence of comorbid systemic diseases.

### Visian implantable collamer lens

The power calculation for the ICL V4c (STAAR Surgical, Nidau, Switzerland) was performed using a modified vertex formula based on the preoperative refractive parameters, according to the manufacturer’s instructions. The size of the implanted ICL V4c was determined from the white-to-white and ACD both obtained using the Pentacam (Pentacam HR, Oculus Optikgeräte GmbH, Wetzlar, Germany) and the recommended size was automatically calculated using the formula provided in the producer’s website (STAAR Surgical, https://ocos.staarag.ch/).

### Surgical procedure

An experienced surgeon (XZ) performed all ICL V4c implantations, as described previously [14]. Briefly, the pupils were dilated preoperatively. A mark was made to indicate the horizontal axis on the limbus to allow toric ICL (TICL) implantation. The ICL was implanted through a 3.0 mm temporal corneal incision with an injector cartridge. A moderate viscoelastic surgical agent (1% sodium hyaluronate) was injected into the anterior chamber, and the ICL V4c was positioned in the posterior chamber. The viscoelastic surgical agent was completely washed out with a balanced salt solution, and a miotic agent was instilled. Postoperative medications included antibiotic, non-steroidal anti-inflammatory, steroidal, and artificial eye drops. Sixty-two eyes were implanted with ICL, including 34 eyes implanted with TICL, which were placed horizontally with a rotation no more than 10°.

### Follow-up examination

All patients underwent preoperative and postoperative ocular examinations. The following main parameters were evaluated: uncorrected and corrected distance visual acuity (UDVA and CDVA, respectively), subjective manifest refraction, intraocular pressure (IOP; Canon, Kanagawa, Japan), corneal topography (Pentacam HR, Oculus Optikgeräte GmbH, Wetzlar, Germany), vault (distance between the posterior ICL surface and anterior crystalline lens surface; AS-OCT, CASIA2) [15], endothelial cell density (ECD; SP-2000P, Tokyo, Topcon Corporation, Japan), and UBM (Quantel Medical, Clermont-Ferrand, France; to assess and exclude abnormal structures of the anterior segment of the eye, such as crystalline lens subluxation, and etc.). Patients were followed up one year postoperatively.

### Lighting conditions

Scotopic, mesopic, and photopic conditions measurements were obtained in a 3-lx (CL-200, KONICA MINOLTA, Tokyo, Japan) dark room (scotopic), with a background light environment of 80 lx (mesopic), and with a penlight, pointed at the temple level of the contralateral eye (photopic).

### Accommodation

Before measurements, we provided + 2.0 D lens to relax the accommodation, gradually increased this to 0.0 D at intervals of − 1.0 D (we defined this non-accommodative stimulus condition as non-accommodation, which may have induced relative accommodation to a certain extent for near accommodation), and then gradually increased this to − 4.0 D (we defined this − 4.0 D accommodative stimulus condition as the accommodation without measuring the actual accommodation) [16, 17]. The patients were asked to specifically focus on the internal fixation target and keep clear of the visual target to achieve an accommodative state. The eye was defined as fully accommodated when the target could no longer be focused on clearly. If the patient was able to keep a clear focus on the visual target continuously under the − 4.0 D accommodative stimulus, images were collected when the pupil was constricted to its minimum. To avoid the induction of accommodation by objects around the target, we performed the measurements in a 3-lx dark room (scotopic) without occluding the fellow eye. Accordingly, we defined the scotopic condition, which was also the non-accommodative stimulus condition, as the baseline. Patients were instructed to avoided reading a book or use their cell phone for 3 h before the measurements [18].

### AS-OCT measurements

The ocular anterior segment parameters were measured by a single ophthalmologist using AS-OCT. Furthermore, the ACD, ACD-ICL (distance from the corneal endothelium to anterior surface of the ICL), vault, pupil diameter, and the crystalline lens tilt value relative to the corneal topographic axis were measured automatically. During photopic, mesopic or accommodation conditions the images with a minimum pupil diameter were selected and during scotopic conditions (baseline) the images with a maximum pupil diameter were selected. Each patient was evaluated three times to exclude interference with image quality, such as eyelash occlusion and inadequate corneal limbus exposure, to achieve optimal image quality for accurate analysis. A total of 128 anterior segment tomographic images (from 0° to 179° with an average interval) of the entire circumference were obtained in 2.4 s. We chose 16 images that display patterns with different directions consisting of 0°, 11°, 23°, 34°, 45°, 56°, 68°, 79°, 90°, 101°, 113°, 124°, 135°, 146°, 158°, and 169° for analysis.

### Tilt values

Images for tilt measurement of the crystalline lens were automatically captured by CASIA 2 (see Additional file 1: Supplementary Method). The measurements of tilt of the ICL V4c has been previously described by Niu et al. [13]. Briefly, the raw measurement images of AS-OCT were exported to MATLAB (R2018a, The MathWorks, Inc., Natick, MA, USA) with a purpose-designed program by Dr He. Four registration lines were manually adjusted to align the anterior and posterior corneal surfaces and ICL surfaces. The location of the marked points on the image were expressed in pixels (X and Y, per pixel equaled to 7.749 μm) relevant to the coordinate XY axis. The corneal topographic axis was defined as the connecting line of the fixation point of the machine and the corneal vertex, which was vertical to the X axis in each image. The tilt value of ICL was determined by averaging the degrees of rotation of the registration lines fitted to the anterior and posterior surfaces of the ICL in each image. The highest value of tilt within the 16 images represented the total tilt value. The horizontal and vertical tilt were the values on the 0- and the 90-degree images. Only absolute values of tilt were used for analysis.

### Statistical analysis

Statistical analysis was performed using R 3.4.3 (http://www.R-project.org). Continuous and categorical variables are expressed as mean ± standard deviation (SD) and frequencies (percentages), respectively. Differences between categorical variables were assessed using the Chi-squared test. The generalized estimating equation model was used to analyze the change in lighting conditions and accommodation, adjusted for both eyes. Different measurement points in both eyes were used as repeated measures variables. Statistical significance was set at *P* < 0.05.

## Results

### Subjects and baseline biometrics

All procedures were successfully performed, and no vision-threatening complications occurred during follow-up. Sixty-two eyes of 31 patients [25 women and 6 men; mean age: 27.90 ± 4.56 years; 95% confidence intervals (CI): 26.23–29.57 years] were enrolled in this study. The safety and efficacy indices (postoperative CDVA/preoperative CDVA and postoperative UDVA/preoperative CDVA) were 1.23 ± 0.18 (95% CI: 1.18–1.27) and 1.11 ± 0.24 (95% CI: 1.04–1.17), respectively. There was no loss of CDVA during follow-up. The one-year postoperative IOP and ECD were 15.21 ± 2.43 mmHg (95% CI: 14.60–15.83 mmHg) and 2559.63 ± 184.17 cells/mm^2^ (95% CI: 2512.86–2606.40 cells/mm^2^), respectively. These values were similar to the preoperative values of 15.18 ± 2.46 mmHg (95% CI: 14.56–15.81 mmHg, *P* = 0.908) and 2,538.42 ± 177.91 cells/mm^2^ (95% CI: 2493.24–2583.60 cells/mm^2^, *P* = 0.368), respectively. The vault was 571.02 ± 251.97 µm (95% CI: 507.03–635.00 µm) under mesopic conditions. No clinically visible subcapsular cataract was observed (Table 1).

**Table 1:** Preoperative and postoperative patient demographic data in eyes implanted with ICL V4c (n = 62 eyes)

Parameters	Mean ± SD	Range (minimum, maximum)
Preoperative		
Age (years)	27.90 ± 4.56	20, 40
CVDA (logMAR)	0.01 ± 0.08	− 0.10, 0.20
Spherical refractive error (D)	− 9.44 ± 2.76	− 6.00, − 18.50
Cylindrical refractive error (D)	− 1.08 ± 0.92	− 3.00, 0.00
SE (D)	− 9.98 ± 2.89	− 6.00, − 18.50
ACD (mm)	3.13 ± 0.21	2.80, 3.47
WTW (mm)	11.58 ± 0.46	10.6, 12.4
IOP (mmHg)	15.18 ± 2.46	10.3, 22.5
ECD (cell/ mm^2^)	2538.42 ± 177.91	2217, 3001
Axial length (mm)	28.30 ± 2.01	24.18, 32.84
ICL size (mm) (median, range)	12.6	12.1, 13.7
Postoperative		
UDVA (logMAR)	− 0.03 ± 0.15	− 0.2, 0.5
CDVA (logMAR)	− 0.09 ± 0.09	− 0.2, 0.2
SE (D)	− 0.13 ± 0.55	− 2.50, 0.25
IOP (mmHg)	15.21 ± 2.43	10.0, 20.8
ECD (cell/ mm^2^)	2559.63 ± 184.17	2206, 3022
Vault (µm)	571.02 ± 251.97	165, 1238

*SD* = standard deviation; *CDVA* = corrected distance visual acuity; *SE* = spherical equivalent; *ACD* = anterior chamber depth; *WTW* = white-to-white; *IOP* = intraocular pressure; *ECD* = endothelial cell density; *UDVA* = uncorrected distance visual acuity; *D* = diopter

### Changes in the three-dimensional position of the ICL V4c

Significant differences were observed in ACD, pupil size, crystalline lens thickness, and anterior surface curvature of the crystalline lens under various lighting conditions and accommodation (*P* < 0.001). The vault did not change during accommodation (*P* = 0.058) but was significantly decreased from scotopic to mesopic and photopic conditions. The ACD-ICL and posterior surface curvature remained unchanged under various lighting conditions (Table 2).

**Table 2:** Parameters of the anterior segment under different lighting conditions and accommodation

Parameters	Baseline (mean ± SD)	Mesopic (mean ± SD)	Photopic (mean ± SD)	*P*	Baseline (mean ± SD)	Accommodation (mean ± SD)	*P*
PD (mm)	5.77 ± 0.97	4.92 ± 0.90 ^a^	3.55 ± 0.79 ^a, b^	0.000	5.77 ± 0.97	5.06 ± 1.23	0.000
LT (mm)	3.75 ± 0.21	3.73 ± 0.21 ^a^	3.77 ± 0.20 ^a, b^	0.000	3.75 ± 0.21	3.85 ± 0.22	0.000
ACD (mm)	3.07 ± 0.18	3.08 ± 0.18 ^a^	3.04 ± 0.18 ^a, b^	0.000	3.07 ± 0.18	2.97 ± 0.20	0.000
Vault (μm)	582.08 ± 269.16	571.02 ± 251.97 ^a^	495.82 ± 263.66 ^a, b^	0.000	582.08 ± 269.16	563.89 ± 269.59	0.058
ACD-ICL (mm)	2.48 ± 0.28	2.51 ± 0.28	2.55 ± 0.28	0.978	2.48 ± 0.28	2.41 ± 0.30	0.000
FC (mm)	10.24 ± 1.27	10.35 ± 1.38 ^a^	9.79 ± 1.45 ^a, b^	0.000	10.24 ± 1.27	9.16 ± 1.39	0.000
PC (mm)	5.73 ± 0.45	5.83 ± 0.69	5.78 ± 0.49	0.364	5.73 ± 0.45	5.54 ± 0.51	0.000

*SD* = standard deviation; *PD* = pupil diameter; *LT* = lens thickness; *ACD* = anterior chamber depth; *ACD-ICL* = distance from corneal endothelium to anterior surface of the ICL V4c; *FC* = front curvature; *PC* = post curvature; ^a^ vs. scotopic; ^b^ vs. mesopic; *P* < 0.05

### Changes in crystalline lens tilt

**Fig. 1:**
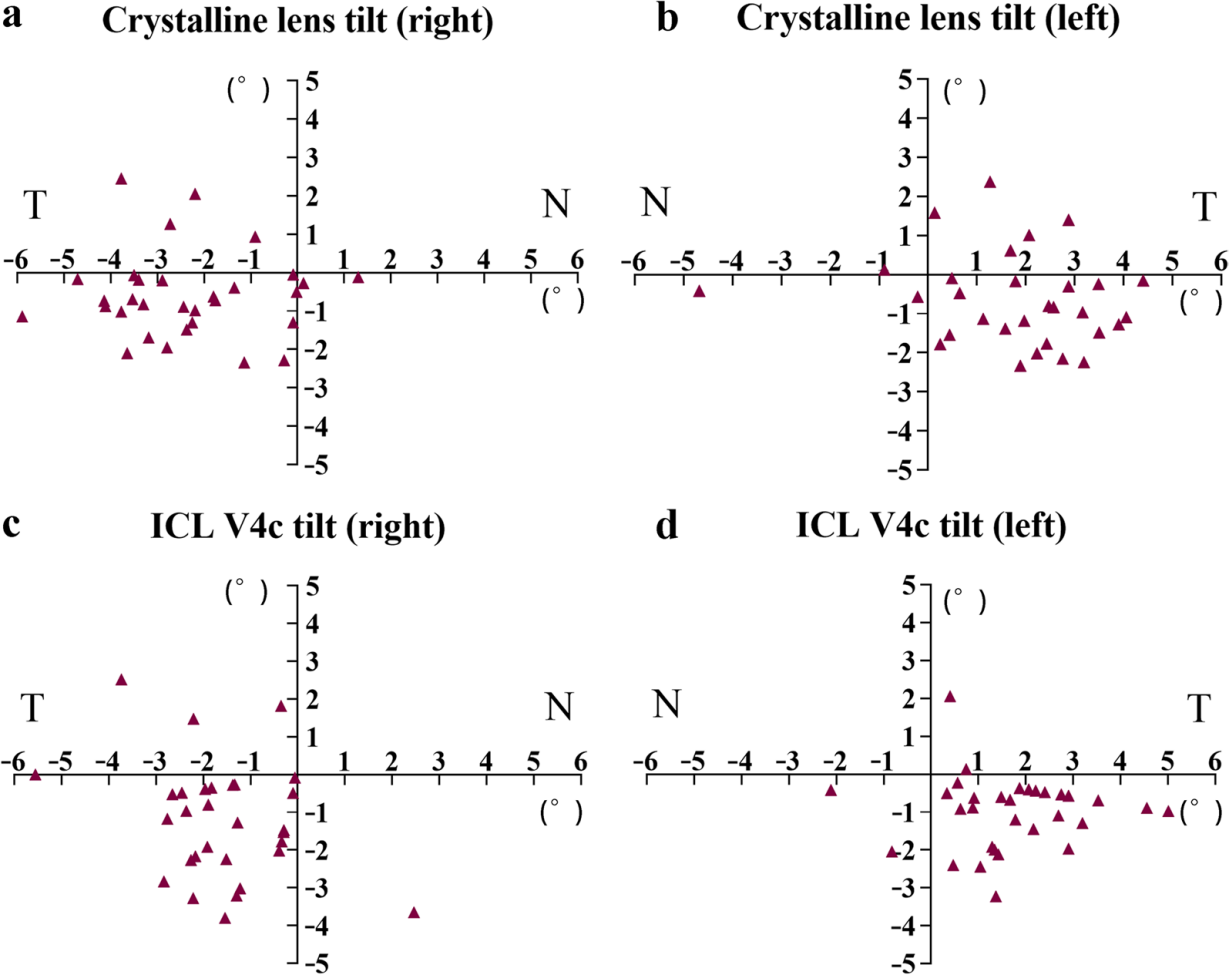
Distributions of crystalline lens and ICL V4c tilt direction of both eyes in mesopic conditions (unit: °). **a** Distributions of crystalline lens tilt direction of right eyes; **b** Distributions of crystalline lens tilt direction of left eyes; **c** Distributions of ICL V4c tilt direction of right eyes; **d** Distributions of ICL V4c tilt direction of left eyes. N, nasal; T, temporal

Under mesopic conditions, the horizontal, vertical, and total tilts of the crystalline lens were 2.32 ± 1.46°, 1.08 ± 0.73°, and 2.76 ± 1.33° (n = 62 eyes), respectively. The crystalline lens was tilted toward the inferotemporal quadrant in 46 eyes (74.2%), inferonasal quadrant in 4 eyes (6.5%), superonasal quadrant in 1 eye (1.6%), and superotemporal quadrant in 11 eyes (17.7%) (Fig. 1).

**Fig. 2:**
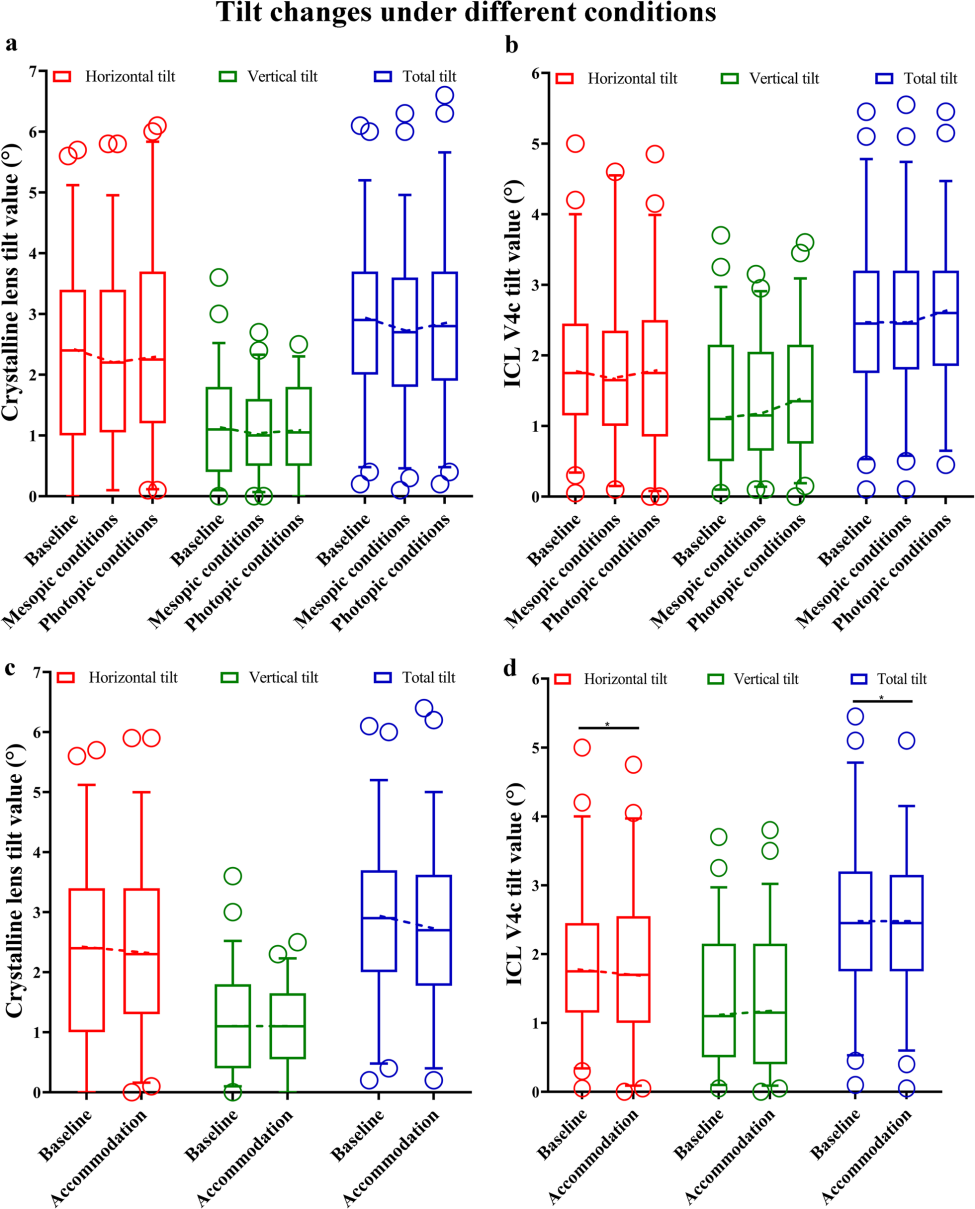
Tilt changes of crystalline lens and ICL V4c under different lighting conditions and accommodation (unit: °). **a** Tilt changes of crystalline lens under different lighting conditions; **b** Tilt changes of ICL V4c under different lighting conditions; **c** Tilt changes of crystalline lens under accommodation; **d** Tilt changes of ICL V4c under accommodation

Among various lighting conditions, no significant differences were observed in horizontal, vertical, and total tilts in the inferotemporal tilted crystalline lens (*P* = 0.245, *P* = 0.463, and *P* = 0.063, respectively). Under the − 4.0D accommodative stimulus state, the horizontal, vertical, and total tilts of the crystalline lens to the inferotemporal quadrant were not significantly different than at baseline (*P* = 0.729, *P* = 0.517, and *P* = 0.415, respectively; Table 3 and Fig. 2). No significant differences were observed in changes in crystalline lens tilts in the superotemporal and inferonasal quadrants among various lighting conditions and during accommodation (all *P* > 0.05, Tables 4 and 5).

**Table 3:** Tilt changes (inferotemporal) of the crystalline lens (n = 46 eyes) and ICL V4c (n = 55 eyes) under different lighting conditions and accommodation

Tilt (°)	Baseline (mean ± SD)	Mesopic (mean ± SD)	Photopic (mean ± SD)	*P*	Baseline (mean ± SD)	Accommodation (mean ± SD)	*P*
Lens horizontal	2.35 ± 1.50	2.30 ± 1.51	2.43 ± 1.63	0.245	2.35 ± 0.50	2.35 ± 1.52	0.729
Lens vertical	1.19 ± 0.79	1.07 ± 0.70	1.12 ± 0.73	0.463	1.19 ± 0.79	1.13 ± 0.68	0.517
Lens total	2.83 ± 1.34	2.75 ± 1.35	2.83 ± 1.39	0.063	2.83 ± 1.34	2.78 ± 1.39	0.415
ICL horizontal	1.86 ± 1.01	1.75 ± 1.09^a^	1.78 ± 1.13	0.667	1.86 ± 1.01	1.75 ± 1.05	0.043
ICL vertical	1.33 ± 0.98	1.34 ± 0.83	1.47 ± 0.91^a^	0.038	1.33 ± 0.98	1.33 ± 0.96	0.215
ICL total	2.51 ± 1.12	2.48 ± 1.11	2.57 ± 1.05	0.072	2.51 ± 1.12	2.36 ± 1.04	0.013

*SD* = standard deviation; ^a^ vs. scotopic; *P* < 0.05

**Table 4:** Tilt changes (inferonasal) of the crystalline lens (n = 4 eyes) and ICL V4c (n = 2 eyes) under different lighting conditions and accommodation

Tilt (°)	Baseline (mean ± SD)	Mesopic (mean ± SD)	Photopic (mean ± SD)	*P*	Baseline (mean ± SD)	Accommodation (mean ± SD)	*P*
Lens horizontal	1.78 ± 1.73	1.68 ± 1.95	1.70 ± 1.49	0.981	1.20 ± 0.00	1.15 ± 0.35	0.874
Lens vertical	0.40 ± 0.24	0.23 ± 0.19	0.23 ± 0.23	0.375	0.35 ± 0.35	0.35 ± 0.35	1.000
Lens total	1.88 ± 1.67	1.80 ± 1.98	1.83 ± 1.65	0.886	1.35 ± 0.07	1.40 ± 0.14	0.500
ICL horizontal	0.95 ± 0.49	1.20 ± 0.78	1.20 ± 0.78	0.374	0.95 ± 0.49	1.60 ± 0.78	0.190
ICL vertical	2.60 ± 0.57	2.65 ± 0.49	2.65 ± 0.49	0.246	2.60 ± 0.57	2.15 ± 0.85	0.266
ICL total	3.15 ± 1.34	3.30 ± 1.56	3.00 ± 1.98	0.500	3.15 ± 1.34	3.15 ± 2.76	1.000

*SD* = standard deviation

**Table 5:** Tilt changes (superotemporal) of the crystalline lens (N = 11 eyes) and ICL V4c (n = 5 eyes) under different lighting conditions and accommodation

Tilt (°)	Baseline (mean ± SD)	Mesopic (mean ± SD)	Photopic (mean ± SD)	*P*	Baseline (mean ± SD)	Accommodation (mean ± SD)	*P*
Lens horizontal	3.10 ± 0.59	3.06 ± 0.61	3.10 ± 0.70	0.991	3.10 ± 0.59	3.06 ± 0.67	0.704
Lens vertical	1.24 ± 0.73	1.54 ± 1.02	1.58 ± 0.79	0.731	1.24 ± 0.73	1.10 ± 0.74	0.643
Lens total	3.38 ± 0.60	3.54 ± 0.67	3.44 ± 0.64	0.999	3.38 ± 0.60	3.40 ± 0.46	0.893
ICL horizontal	2.19 ± 0.63	1.94 ± 0.66	2.17 ± 0.55	0.907	2.19 ± 0.63	2.31 ± 0.74	0.395
ICL vertical	2.37 ± 1.25	2.12 ± 1.11	1.72 ± 0.89	0.932	2.37 ± 1.25	2.42 ± 1.04	0.941
ICL total	2.53 ± 1.41	2.37 ± 1.38	2.13 ± 0.91	0.968	2.53 ± 1.41	2.38 ± 1.65	0.807

*SD* = standard deviation

### Changes in ICL V4c tilt

Under mesopic conditions, the average ICL V4c tilt averaged at 1.75 ± 1.05° horizontally, 1.44 ± 0.89° vertically, and 2.50 ± 1.13° in total tilt (n = 62 eyes). In total, 55 eyes (88.7%), 2 (3.2%), and 5 (8.1%) exhibited inferotemporal, inferonasal, and superotemporal ICL V4c tilt, respectively (Fig. 1). The inferotemporal proportion of ICL V4c and crystalline lens tilts were similar (*P* = 0.063).

There was no statistically significant difference between the vault and tilt of eyes implanted TICL or ICL under different lighting conditions and accommodation. From baseline to photopic conditions, we observed a significant increase in vertical tilt of ICL V4c tilted to the inferotemporal quadrant (n = 55 eyes; *P* = 0.038), while the horizontal (*P* = 0.667) and total (*P* = 0.072) tilts were similar. Under the − 4.0 D accommodative stimulus state, the horizontal (*P* = 0.043) and total (*P* = 0.013) tilts to the inferotemporal quadrant were significantly lower than baseline, while the vertical tilt was not (*P* = 0.215; Table 3 and Fig. 2). No significant differences were observed in the ICL V4c tilt changes in the inferonasal and superotemporal quadrants under various lighting conditions and accommodation (all *P* > 0.05, Tables 4 and 5).

## Discussion

To the best of our knowledge, this is the first study to use AS-OCT to evaluate the three-dimensional position, including axial shifts and tilt, of the crystalline lens and ICL V4c under various lighting conditions and accommodation.

We observed small axial shifts of the ICL V4c during accommodation. The ACD and ACD-ICL, but not vault, were reduced under the accommodation stimulus of − 4.0 D. According to Helmholtz theory, the anterior lens pole moves forward and the ciliary body contracts during accommodation, and thus reduces the ciliary ring diameter. The four haptics of the ICL V4c are in the ciliary sulcus. Contraction of the ciliary muscle causes the anterior movement of the crystalline lens and the axial shift of the ICL V4c toward the corneal endothelium, resulting in an insignificant reduction in the vault. This is consistent with the results of Lindland et al. [10] who used Visante OCT and observed no difference in the vault between the baseline and the accommodation states. However, Du et al. [8] reported that pharmacologic accommodation caused pupillary sphincter and ciliary muscle contraction, leading to a decrease in the ICL V4c vault.

Our results revealed a slight decrease in the ACD and vault from the scotopic to the photopic condition, with no change in the ACD-ICL. Petternel et al. [19] employed a penlight shining into the contralateral eye, while Kimira et al. [15] used background light under dynamic observation. Both studies reported a decrease in the vault, which was in line with our results. The lack of changes in the ACD-ICL implies that the axial position of the ICL V4c in the anterior chamber remains stable under the various lighting conditions.

Under mesopic conditions, 74.2% of the crystalline lens and 88.7% of the ICL V4c were tilted toward the infratemporal quadrant, similar to results reported by Kimura et al. [20] for crystalline lens tilted toward the infratemporal quadrant relative to the corneal topographic axis under non-dilated conditions, and to the crystalline lens tilted 2.85° with the anterior crystalline lens surface facing the inferotemporal quadrant, as reported by Hu et al. [21]. However, the total crystalline lens tilt (2.76 ± 1.33°) was smaller than previously reported (i.e., 5.15°) [20]. The differences in tilt degree could be underscored by age differences, as the mean age in that study was 73.6 years (range, 44 to 90 years), whereas the maximum age in our study was 40 years, with a mean of 27.90 ± 4.56 years. Mester et al. [22] used a Purkinje meter and observed that the crystalline lens tilt position in young individuals was more centered, with a mean horizontal tilt of 3.1° and vertical tilt of 2.2°. A tilt of the IOL toward the infratemporal quadrant after cataract surgery has been reported [20]. However, the symmetry of the postoperative horizontal tilt was disrupted, possibly due to the non-mirror symmetry in the incision positions in both eyes, as the authors performed temporal and nasal incisions in the right and left eye, respectively. In contrast, all patients in our study underwent mirror-symmetrical incisions at the temporal 3 o’clock position; hence, the postoperative tilt direction remained highly symmetrical in both eyes.

Under the non-accommodation conditions [23], the ciliary muscle in rhesus monkeys has been reported on the temporal side rather than on the nasal side, which may explain the tilt of most crystalline lens tilt toward the temporal side. Nasal temporal anatomic variations have been suggested to be a functional necessity in primates, enabling maintenance of binocular single vision during the convergent eye movements that accompany accommodation [24]. In this regard, the infratemporal tilt of the crystalline lens or the ICL V4c is consistent with the physiological function of the eye. We did not observe a significant difference in the crystalline lens tilt under various lighting and accommodative states, indicating that the crystalline lens tilt remained stable under these conditions.

This is the first report on tilt changes under various lighting conditions and accommodation after ICL V4c implantation. The vertical tilt of the ICL V4c increased from baseline to the photopic conditions because the haptics of the ICL V4c are fixed in the ciliary sulcus, while the anterior surface of ICL V4c is in contact all around with the posterior surface of the iris [8]. The increase in the ICL V4c vertical tilt during pupillary constriction in the mesopic and photopic conditions could be due to asymmetrical iris compression force in the vertical direction. Our results are consistent with previous findings that demonstrated a significant increase in IOL tilt along the horizontal axis during accommodation, approximating to the hinged IOL haptics orientation [25].

The horizontal and total tilts were lower during accommodation than at baseline. The ICL V4c was subjected to the ciliary muscle force on the four haptics. The ICL V4c was placed in a horizontal orientation in all patients in this study, and the haptic positions were primarily in the horizontal direction above and below the nasal and temporal sides. Contraction of the ciliary muscle during accommodation exerts great force in the horizontal orientation. During accommodation, shortening of the thicker temporal ciliary muscle may result in a stronger contractile response than that on the nasal side, with highly pronounced thickening of the anterior ciliary muscle [24]. Furthermore, most of the ciliary muscle weight shifts forward and inward during accommodation to reduce tension on the suspensory ligament, and this action is more pronounced on the temporal side. These phenomena may drive the asymmetric movement of the ICL V4c and reduce the tilt toward the temporal side. We did not find statistical significant difference between the vault and tilt of eyes implanted TICL or ICL under different lighting conditions and accommodation. Lindland et al. [26] found a similar change of the vault during accommodation in eyes with ICL and TICL; however, the tilt change deserves further study.

This study had several limitations. First, there was only one postoperative observation point. Kojima et al. [27] reported that the vault remained stable one year after ICL V4c surgery. The patients in our study were followed up for one year postoperatively and exhibited consistent findings. Further research is needed to examine the longitudinal comparison of the tilt at different time points. Second, not all the TICL were precisely implanted at the 180° meridian. Nevertheless, all TICLs were placed horizontally with a rotation no more than 10° which was to reduce the potential impact on the tilt. However, even a small rotation may influence the results, which warrants further verification. Third, the accommodative stimulus of − 4.0 D was provided in the study, but the amount of accommodation induced in each participant could not be measured. Further investigation is warranted regarding the relationship between the tilt change and the accommodation. Additionally, further studies are needed for pharmacologic mydriasis to achieve a completely non-accommodative station. At last, though the penlight was fixed on both sides of the machine at the temple level of the eye and the distance, direction, and luminance of the penlight may be equal, the precise lux of each time may not be reproducible. Further study is warranted using fixed lux illumination conditions.

## Conclusion

The axial position of the ICL V4c in the anterior chamber was stable under various lighting conditions. The vertical and horizontal tilts of ICL V4c may be particularly influenced by lighting conditions and accommodation, respectively.

## Supplementary Information

**Additional file 1: Figure S1**. Tilt measurement of crystalline lens automatically captured by CASIA 2. The two-dimensional image shows the anterior and posterior views of the cornea (lines a and b) and the anterior and posterior curvature of the crystalline lens (lines c and d), which are extended to intersect at two symmetrical points and thus the outline of the crystalline lens is obtained (crystalline lens axis, yellow line; corneal topography axis, blue line). Tilt of the crystalline lens is defined as the angle between the crystalline lens axis and the corneal topography axis. **Figure S2**. Four registration dotted lines from the top to the bottom are aligned to the anterior (line a) and posterior (line b) surfaces of the cornea and the anterior (line c) and posterior (line d) surfaces of the ICL V4c. For registration, all four dotted lines can be moved (horizontally and vertically), and lines c and d can be rotated (clockwise or anti-clockwise) and their curvature radius can be changed by clicking relevant buttons in the software. The blue dashed line (line e) represents the vertical line passing through the corneal vertex. **a** Line a and line b have been aligned exactly to the anterior and posterior surfaces of the cornea. Alignment of lines c and d before rotation and changed by clicking relevant buttons in the software. **b** Alignment of lines c and d after clockwise rotation and changed. The tilt value of ICL V4c was determined by averaging the degrees of rotation of the registration lines c and d fitted to the anterior and posterior surfaces of the ICL V4c in each image.

## Data Availability

The data that support the findings of this study are available from the corresponding author upon reasonable request.

## Abbreviations

ACD: Anterior chamber depth
ACD-ICL: Distance from the corneal endothelium to anterior surface of the ICL V4c
AS-OCT: Anterior segment optical coherence tomography
CDVA: Corrected distance visual acuity
ECD: Endothelial cell density
ICL V4c: Visian implantable collamer lens
IOL: Intraocular lens
IOP: Intraocular pressure
TICL: Toric ICL
UBM: Ultrasound biomicroscopy
UDVA: Uncorrected distance visual acuity
