# Supplementary material for: Effects of lighting conditions and accommodation on the three-dimensional position of Visian implantable collamer lens
Source: Eye Vis (Lond). 2022 Nov 4;9:42. doi: 10.1186/s40662-022-00313-2 (PMC9635130; doi:10.1186/s40662-022-00313-2)

**Supplementary**

**Figure S1**. Tilt measurement of crystalline lens automatically captured by CASIA 2. The two-dimensional image shows the anterior and posterior views of the cornea (lines a and b) and the anterior and posterior curvature of the crystalline lens (lines c and d), which are extended to intersect at two symmetrical points and thus the outline of the crystalline lens is obtained (crystalline lens axis, yellow line; corneal topography axis, blue line). Tilt of the crystalline lens is defined as the angle between the crystalline lens axis and the corneal topography axis.


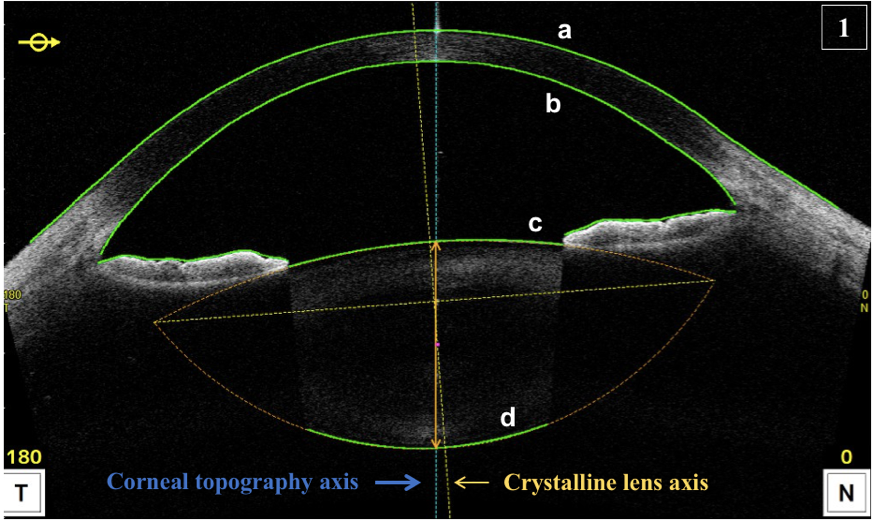


**Figure S2**. Four registration dotted lines from the top to the bottom are aligned to the anterior (line a) and posterior (line b) surfaces of the cornea and the anterior (line c) and posterior (line d) surfaces of the ICL V4c. For registration, all four dotted lines can be moved (horizontally and vertically), and lines c and d can be rotated (clockwise or anti-clockwise) and their curvature radius can be changed by clicking relevant buttons in the software. The blue dashed line (line e) represents the vertical line passing through the corneal vertex. **a** Line a and line b have been aligned exactly to the anterior and posterior surfaces of the cornea. Alignment of lines c and d before rotation and changed by clicking relevant buttons in the software. **b** Alignment of lines c and d after clockwise rotation and changed. The tilt value of ICL V4c was determined by averaging the degrees of rotation of the registration lines c and d fitted to the anterior and posterior surfaces of the ICL V4c in each image.


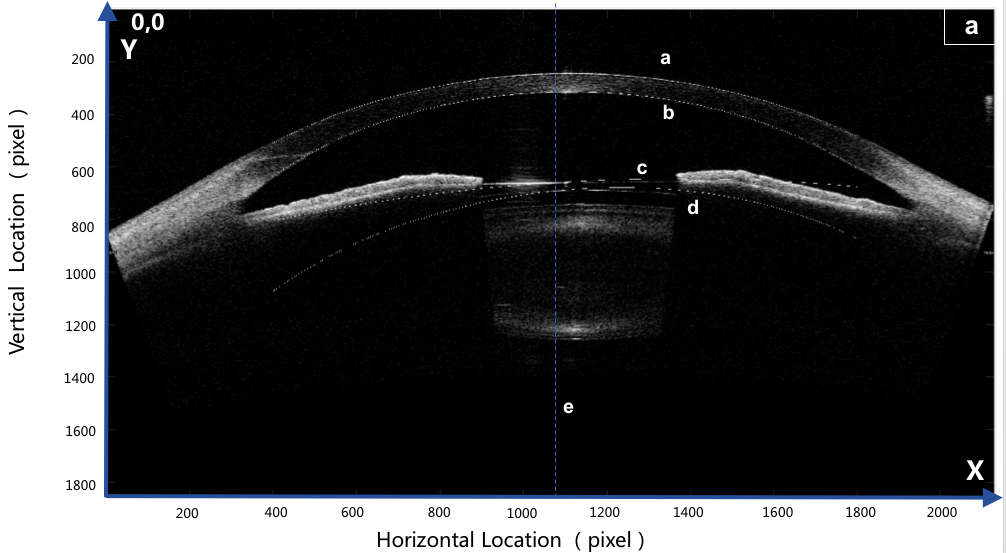


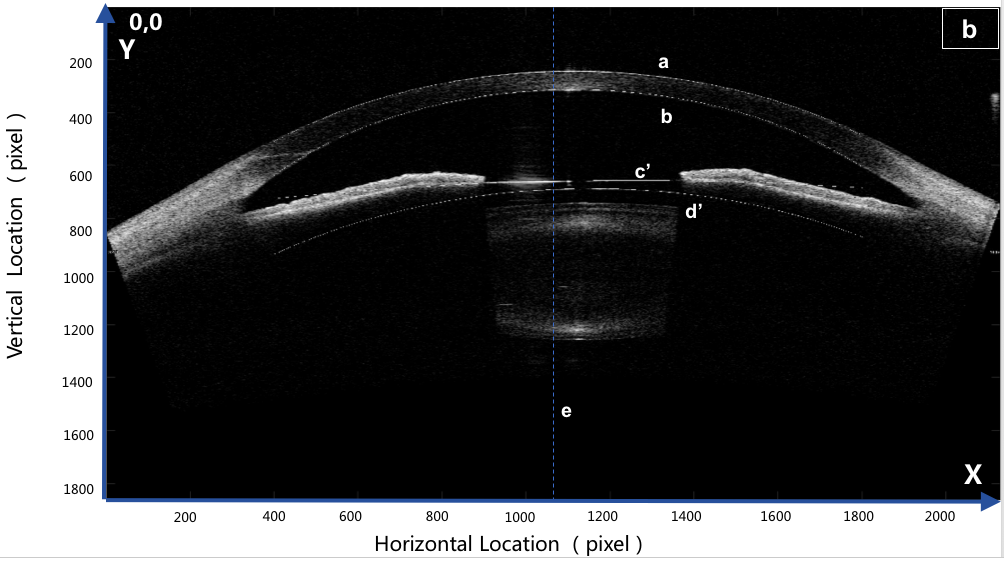

Supplement: Supplementary file 1 — Additional file 1: Figure S1. Tilt measurement of crystalline lens automatically captured by CASIA 2. The two-dimensional image shows the anterior and posterior views of the cornea (lines a and b) and the anterior and posterior curvature of the crystalline lens (lines c and d), which are extended to intersect at two symmetrical points and thus the outline of the crystalline lens is obtained (crystalline lens axis, yellow line; corneal topography axis, blue line). Tilt of the crystalline lens is defined as the angle between the crystalline lens axis and the corneal topography axis. Figure S2. Four registration dotted lines from the top to the bottom are aligned to the anterior (line a) and posterior (line b) surfaces of the cornea and the anterior (line c) and posterior (line d) surfaces of the ICL V4c. For registration, all four dotted lines can be moved (horizontally and vertically), and lines c and d can be rotated (clockwise or anti-clockwise) and their curvature radius can be changed by clicking relevant buttons in the software. The blue dashed line (line e) represents the vertical line passing through the corneal vertex. a Line a and line b have been aligned exactly to the anterior and posterior surfaces of the cornea. Alignment of lines c and d before rotation and changed by clicking relevant buttons in the software. b Alignment of lines c and d after clockwise rotation and changed. The tilt value of ICL V4c was determined by averaging the degrees of rotation of the registration lines c and d fitted to the anterior and posterior surfaces of the ICL V4c in each image. [file 40662_2022_313_MOESM1_ESM.docx]
